# Omicron (B.1.1.529): constellation of unusual mutations in SARS-CoV-2

**DOI:** 10.4314/ahs.v23i2.2

**Published:** 2023-06

**Authors:** Abhishek Kashyap, Indrajit Banerjee

**Affiliations:** Sir Seewoosagur Ramgoolam Medical College, Belle Rive, Mauritius

**Dear Sir,**

On 26^th^ November 2021, the World Health Organization classified SARS-CoV-2 variant B.1.1.529 as the fifth variant of concern (VOC), after the delta variant, and labeled it “Omicron.” It could be the first indicator considering it a very high risk for public health amidst the vaccine hesitancy observed in several countries. 1

This variant was sequenced in the specimen collected from 14^th^ to 23rd November in South Africa and 11^th^ November in Botswana. 2,3 As of 11^th^ June, 21:00 GMT, 15,832 confirmed cases had been reported in various provinces of South Africa, which strongly indicates this variant drove the fourth wave. First positive cases were noticed in Tshwane and Gauteng Province. In the following few days, KwaZulu-Natal and Western Cape provinces were also positive for this lineage; consequently, several countries, including European Union, have implemented an emergency brake mechanism. 2,3,4

The Omicron variant is one of the most divergent variants detected so far, which was later revised into three sub lineages- BA.1, BA.2, and BA.3. More than 15 mutations have already been identified in the spike receptor-binding domain, along with four amino acids substitutions, three small deletions, and one small insertion in the N-terminal domain. Omicron also has three mutations in the S1/S2 Furin cleavage site- H655Y, P681H & N679K, detected for the first time in a single variant. Another deletion in nsp6 outside the spike protein and double mutation in nucleocapsid has also been detected. This detrimental change in COVID-19 epidemiology is estimated to have a growth advantage of 0.24 per day over the delta variant. 3,5 The variant can be detected with – S gene dropout or S gene target Failure during TaqPath- based PCR testing, which was used as a marker for identification. However, this is not a characteristic marker for identification because the alpha variant also has the same mutation. Evidence indicates a heightened risk of re-infection, transmissibility, immune escape, and reduced efficiency of vaccines due to alternation in the spike protein, which is the primary antigenic target for most of the authorized monoclonal antibodies and vaccines. 5,6 According to epidemiologists, two reported cases in Hong Kong were fully vaccinated with the Pfizer vaccine six months back. 1,3,4 Several studies suggest administering a booster dose for better vaccine response. 6 Currently, no new symptoms were identified to differentiate it from earlier variants. 4

This variant poses a high risk to the global vaccination regime, while the world faces a gross vaccine inequity, a rising infection rate, and a fractionally vaccinated population. We are actively moving ahead with the evolution of this virus to our prejudice unless we give utmost priority to vaccination and containment of the virus. Close monitoring of Omicron in South Africa will be necessary to understand its transmission dynamics better.^3^

## Limitations

This article is an exploratory attempt to discuss the nature of the Omicron variant, basis the limited publicly accessible data and scientific articles on this issue. Several aspects of virological characterization, epidemiological analysis, assessment of the severity of this strain, vaccine effectiveness, and sequencing was not included due to the unavailability of data.
